# Socio-demographic and substance-related factors associated with mental distress among Wollo university students: institution-based cross-sectional study

**DOI:** 10.1186/s12991-019-0252-4

**Published:** 2019-12-21

**Authors:** Yosef Zenebe, Mogesie Necho

**Affiliations:** Department of Psychiatry, College of Medicine and Health Sciences, Wollo University, Dessie, Ethiopia

**Keywords:** Mental distress, Wollo university, Dessie, Ethiopia

## Abstract

**Background:**

The presence of mental distress among students affects their cognitive, emotional, physical, and interpersonal functioning. Besides, it predisposes to substance use problems and finally affects academic performance negatively. Therefore, this study was designed to estimate the magnitude and identify associated factors for the mental distress of students at Wollo University, Dessie, Ethiopia.

**Methods:**

This institution-based cross-sectional survey assessed mental distress among 585 undergraduate students at Wollo University from April 10 to May 10/2019 using a multi-stage stratified sampling technique. Kessler-10 item scale was used to collect data about mental distress. Variables with *p*-value < 0.25 in bivariate logistic regression were pooled into a multi-variable logistic regression model and *p*-value < 0.05 in the multi-variable model was considered significant statistically. The strength of the relationship was interpreted using the odds ratio with 95% CI. The model adequacy in multi-variable regression was approved with Hosmer and Lemeshow goodness of fit test.

**Results:**

Among 585 questionnaires distributed, 548 clear and completed questionnaires were included in the analysis with a response rate of 93.7%. The mental distress prevalence in the current study was 106 (19.3%). From this 65 (11.9%), 28 (5.1%), and 13 (2.4%) were found to be mild, moderate and severe mental distress respectively. Never attending a place of worship (AOR = 4.2, 95% CI 1.73, 10.39), family history of mental illness (AOR = 2.1, 95% CI 1.12, 3.95), current cigarette smoking (AOR = 3.2, 95% CI 1.69, 6.20), current alcohol use (AOR = 2.5, 95% CI 1.49, 4.25), and current cannabis use (AOR = 3.4, 95% CI 1.18, 9.57) were the associated factors for mental distress.

**Conclusion:**

One in five students was affected by mental distress. Never attending a place of worship, family history of mental illness, current cigarette smoking, current alcohol use, and current cannabis use were the factors associated with it. Therefore, all stakeholders should be involved in overcoming this public health problem. Besides, clubs should be established in the university and have to play an active role in bringing behavioral change to substance use.

## Background

Mental distress can be defined as a state of mind with vague manifestations of anxiety, depression as well as ranges of somatic symptoms like headache, backache, and disturbance of vegetative functions such as disturbed sleep [1, 2]. The contribution of mental disorders like mental distress to the burden of diseases and disability is alarmingly increasing globally. The latest reports from World Health Organization (WHO) showed that nearly 33.3% of the disability-adjusted life years in the world is due to psychiatric disorders of different type and of all population in the world, one in four individual is affected by a mental disorder [3]. Though little attention is given, African countries also take the highest share of mental distress and the contribution of mental distress to the burden of disease and disability in Africa has been documented to be 5% and 19%, respectively [4].

University students are at a higher risk of mental distress when compared to the general population [5] or their community counterparts [6–8] and the prevalence of mental distress in students varies widely in different countries and study periods. The Worldwide prevalence of mental distress among medical students was documented to be in a range of 25–90% [9, 10]. A study at Fayoum University showed that 62.4%, 64.3%, and 60.8% of students were found to have mental distress, anxiety, and depression, respectively [11]. Many other studies also revealed that mental distress was 39.7% (20.7% mild, 7.8% moderate, and 11.2% severe distress) in Canada [12], 50% in Singapore [13], 70% in Pakistan [14], 19.2% and 16.5% in Australia [8, 15], 41% in Malaysia [16], 52% in India [17] and 26.9% in Nigeria [18], 19.8% in Somaliland, 40.9%, 30% and 39.6% in Ethiopia [19–21].

Many factors were identified so far that contribute to a higher prevalence of mental distress in university students [22–26]. A study in France justified that female gender, alcohol abuse, smoking cigarette, and cyber addiction were contributing risks for mental distress [27]. Other studies also showed that a family history of mental illness was an associated factor for mental distress in students in Iraq [10], Pakistan [14], and Ethiopia [19, 28]. Besides, substance-related variables in France [27], Nepal [29] and Somaliland [30], Female gender in United States [26], Iran [10], Sweden [31], Australia [8] and Somaliland [30], poor worship practice in Ethiopia [19, 28] were some of the factors identified to account for mental distress in university students.

Mental distress affects many aspects of a student's life such as problems of interpersonal relationship, cognitive, physical, and emotional dysfunction, and inability to attain and sustain pleasure in life in many ways as well as increased test anxiety and poor-self-efficacy. This, in turn, will have a detrimental impact on the ability of students to study and finally will end in poor academic achievement; lowers student grades [8, 31, 32]. Above all, mental distress in students if not addressed timely will progress to more severe psychiatric disorders. Despite this, there is a poor help-seeking intention for mental distress among university students [33–35].

However, little has been done in this area among university students in Ethiopia; even those studies done focused particularly on medical students [20, 36] and it might not be representative of other university students. Moreover, some of the studies are done in the long past and might not be consistent with the changing situation of university students at present. The aim of this study was therefore to assess the prevalence and factors associated with mental distress among students in Wollo University.

## Methods and materials

### Study design and setting

This institution-based cross-sectional survey was aimed to assess the magnitude of mental distress and identify the factors associated with it among undergraduate students in a regular study at Wollo University from April 10 to May 10/2019. This study was conducted at Wollo University, Dessie, Amhara Regional State, 400 km from the capital city of Ethiopia; Addis Ababa, in the Northeast of Ethiopia. Dessie is a low temperate area and its latitude and longitude location are 11°8′N and 39°38′E, respectively with an elevation between 2470 and 2550 m above sea level [37]. Wollo University, Dessie campus has 5 colleges, 2 schools and a total of 62 departments.

### Participants

The number of undergraduate regular students enrolled in 2018/2019 in Dessie campus colleges, schools, and departments of Wollo University is 7248 and it was considered as the source population whereas the study population consisted of all undergraduate regular students from eighteen selected departments at final stage of multi-stage sampling and enrolled in 2018/ 2019 in the selected colleges. All students from selected departments present at the time of data collection were included while those who were absent and with impairment in the visual field were excluded.

The required sample size for this study was calculated using proportion formula for a single population and was found to be 585. The following assumptions were considered in the calculation procedure; Prevalence of mental distress among university students 41% from a previous study in Gondar University [19], the level of significance to be within 5% margin of error and 95% confidence level, correction formula has been used since the number of regular undergraduate students in 2019 at Wollo university (N) was 7248 which is < 10,000, 10% contingency for probable non-response and a design effect of 1.5 has been taken as multiple stages were involved in calculating sample size.

Multi-stage stratified sampling was adopted for this study according to the colleges and year level of the students. In the first stage, we selected randomly three colleges (college of medicine and health sciences, college of law and College of Natural science) using a lottery method. In the second stage, again lottery method was employed to select 18 departments from three colleges selected at first stage and finally, the proportional allocation was used to determine the number of students to be included from each department and study years.

### Assessment of mental distress and its independent variables

Data were collected using structured pre-tested and self -administered questionnaires in the English language. The first section is the questionnaire for the assessment of socio-demographic (age, sex, residence, frequency of worship practice, and family history of mental illness) and academic-related variables (department choice, interest to one’s department, year of study, cumulative grade point average, and having a close friend at school) which were developed by investigators by reviewing previously published works.

The next section was the Kessler-10 psychological distress scale which is a measurement scale for mental distress [38]. This scale asses the following 10 elements in the past 4 weeks (About how often did you feel tired out for no good reason? about how often did you feel nervous? about how often did you feel so nervous that nothing could calm you down?, about how often did you feel hopeless?, about how often did you feel restless or fidgety?, about how often did you feel so restless you could not sit still?, about how often did you feel depressed?, about how often did you feel that everything was an effort?, about how often did you feel so sad that nothing could cheer you up? and about how often did you feel worthless?) each element with a five-level response scale ranging from score1 (none of the time) to score 5 (all of the time). Scores of the 10 elements are then summed, providing a minimum total score of 10 and a maximum score of 50.

A score of 10–19 was considered normal, 20–24 likely to have mild distress, 25–29 a moderate distress, and 30–50 was considered as severe distress with an overall score of 20 suggesting mental distress [38]. The internal consistency of the Kessler-10 psychological distress scale in the present study was checked with a reliability assessment and was found to be 0.86. This scale has been utilized in many previous studies in Ethiopia [39–41]. The last section consisted of questions for the assessment of substance-related variables (khat, alcohol, cigarette, and cannabis) [42]. Data collectors were trained BSc nurses and two MSc in mental health professionals and the principal investigator were closely monitoring the data collection process. A pre-test has been implemented on 28 regular undergraduate students a week before the main data collection period and the result was not part of the final survey.

### Data management methods

After the distributed questionnaires were collected back, the task done was exploring and checking for its completeness as well as data cleaning. Consequently, complete data were entered to epi-info version-7 and analyzed using SPSS version 20 software package. Descriptive statistics were done to illustrate the distribution of socio-demographic, academic and substance use characteristics of the study participants. The association of mental distress with its independent variables was assessed using binary logistic regression. All independent variables with *p*-value < 0.25 in bivariate logistic regression were pooled into a multi-variate logistic regression model and *p*-value < 0.05 in the final model was considered statistically significant.

The strength of the relationship between mental distress and its correlates was interpreted using AORs with 95% CI. The model adequacy in final multi-variable regression was approved with Hosmer and Lemeshow goodness of fit test.

### Ethical considerations

This research was conducted after obtaining an official ethical clearance from the Ethical institutional review committee of Wollo University. An official letter from Wollo University Psychiatry department was written to the university academic vice president office to get permission for data collection. Finally, a permission letter from the university academic vice president office was received. Purpose and importance of the study were explained to the study participants and written informed consent was obtained from participants before starting the data collection. Confidentiality of the data was assured by keeping anonymous procedures throughout the research work.

## Results

### Socio-demographic and academic-related characteristics of Wollo university regular undergraduate students

Out of the total 585 questionnaires distributed to students for data collection, 548 were collected back and responded with complete data making the response rate to be 90.9%. The mean (± SD) age of the participant students was 21.4 (± 1.82) years and more than half (62.6%) of the students were above mean age. Nearly half, 291 (53.1%), were males. About 343 (62.6%) of participants have satisfactory results based on the CGPA score. The proportion of students in the first, second, third and fourth year were 33.8%, 26.5%, 31.9, and 8%, respectively. The majority of students, 441 (80.5%), have close friends and 70 (12.8) have a history of mental illness. One hundred twenty-five (22.8%) of participated students joined their department without their choice and 82 (15%) are not interested in their department currently (Table 1).

**Table 1:** Description of socio-demographic and academic-related characteristics among Wollo university regular undergraduate students, Dessie, Ethiopia, 2019 (*n* = 548)

Independent variables	Category	Frequency (n)		Percentage (%)
Age in years	≤ 21 years	205		37.4
	> 21 years	343		62.6
Sex	Male	291		53.1
	Female	257		46.9
Department choice	Preferred	423		77.2
	Not preferred	125		22.8
Interest to their department	Interested	466		85.0
	Not interested	82		15.0
Year of study	First	184		33.6
	Second	145		26.5
	Third	175		31.9
	Fourth	44		8.0
CGPA	Satisfactory	343		62.6
	Distinction	57		10.4
	Great Distinction	94		17.2
	Very Great Distinction	54		9.9
Living area	In the compound	496		90.5
	Out of the compound	50		9.1
Residence	Urban	304		55.5
	Rural	244		44.5
Frequency of worship practice	Daily	245		44.7
	2–3 times per week	144		26.3
	Once a week	70		12.8
	Less than weekly	59		10.8
	Never	30		5.5
Having close friends	Yes	441		80.5
	No	107		19.5
Family history of mental illness	Yes	70		12.8
	No	478		87.2

*CGPA* cumulative grade point average

### Substance-related characteristics of Wollo university regular undergraduate students

From all university students participated in current study, 139 (25.4%) have been using alcohol in the past one month, 104 (19%) have been using khat, and 22 (4%) had been entertained in using cannabis (hashish) during the last one month of study period (Table 2).

**Table 2:** Substance use characteristics of Wollo university regular undergraduate students, Dessie, Ethiopia, 2019 (*n* = 548)

Independent variables	Category	Frequency (n)	Percent (%)
Current khat use	Yes	104	19.0
	No	444	81.0
Current cigarette use	Yes	62	11.3
	No	486	88.7
Current alcohol use	Yes	139	25.4
	No	409	74.6
Current cannabis use	Yes	22	4.0
	No	526	96.0

Current khat use: use of khat in the last one month

Current cigarette use: smoking cigarette in the last one month

Current alcohol use: use of beer, tella, areki, or wine in the last one month

Current cannabis use: use of cannabis (hashish) in the last one month

### Prevalence of mental distress among Wollo university regular undergraduate students

**Fig. 1:**
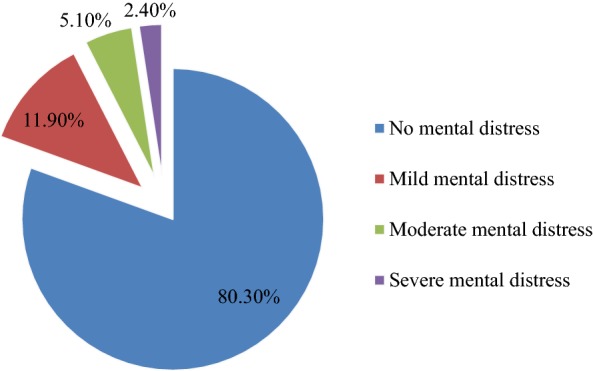
Prevalence of mental distress among Wollo university regular undergraduate students

Mental distress among students in this study was assessed with Kessler-10 mental distress scale with a score of 20–24, 25–29, and 30–50 indicating mild, moderate, and severe mental distress respectively which means that a score of 20 and above on Kessler-10 mental distress scale indicates mental distress and a score of less than 19 on Kessler-10 was considered as not having mental distress. The prevalence of mental distress among Wollo university undergraduate students in this study was 106 (19.3%) with 95% CI (16.10, 22.80). From this, 65 (11.9%), 28 (5.1%), and 13 (2.4%) of participants were found to have mild, moderate, and severe mental distress, respectively (Fig. 1). The mean value of prevalence of mental distress in this study was 12.94 with a standard deviation (SD) and variance of 8.18 and 66.99, respectively.

**Fig. 2:**
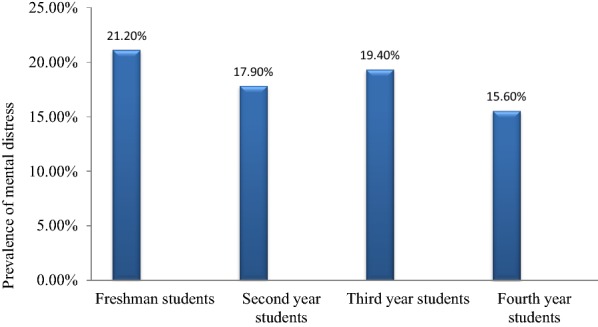
Prevalence of mental distress as the seniority level of students increases

The prevalence of mental distress among males is 19.6% and females 19% but this difference is not statistically supported (AOR = 1.46 95% CI (0.86, 2.47). The highest prevalence of psychological distress was noticed among freshman students (Fig. 2). Considering specific mental distress symptoms about 112 (20.4%), 77 (14.1%), and 72 (13.1%) of students feel that everything was an effort, feel restless/fidgety, and feel depressed, respectively most of the time in the past 4 weeks of data collection (Table 3).

**Table 3:** One month Prevalence of mental distress symptoms among Wollo University regular undergraduate students, Dessie, Ethiopia, 2019 (*n* = 548)

No	Feeling symptom domain in the past 4 weeks (Kessler-10 elements)	Frequency (%) of participants with the symptom				
		All of the time (score 5)	Most of the time (score 4)	Some of the time (score 3)	A little of the time (score 2)	None of the time (score 1)
1	About how often did you feel tired out for no good reason?	28 (5.1%)	66 (12%)	136 (24.5%)	167 (30.5%)	151 (27.6%)
2	About how often did you feel nervous?	19 (3.5%)	58 (10.6%)	145 (26.5%)	147 (26.8%)	179 (32.7%)
3	About how often did you feel so nervous that nothing could calm you down?	24 (4.4%)	50 (9.1%)	112 (20.4%)	152 (27.7%)	210 (38.3%)
4	About how often did you feel hopeless?	26 (4.8%)	45 (8.2%)	102 (18.6%)	159 (29.0%)	216 (39.4%)
5	About how often did you feel restless or fidgety?	33 (6%)	77 (14.1%)	118 (21.5%)	153 (27.9%)	167 (30.5%)
6	About how often did you feel so restless you could not sit still?	28 (5.1%)	63 (11.5%)	130 (23.7%)	136 (24.8%)	191 (34.9%)
7	About how often did you feel depressed?	22 (4%)	72 (13.1%)	123 (22.4%)	175 (31.9%)	156 (28.5%)
8	About how often did you feel that everything was an effort?	62 (11.3%)	112 (20.4%)	118 (21.5%)	117 (21.4%)	139 (25.4%)
9	About how often did you feel so sad that nothing could cheer you up?	8 (1.5%)	53 (9.7%)	123 (22.4%)	200 (36.5%)	164 (29.9%)
10	About how often did you feel worthless?	25 (4.6%)	63 (11.5%)	101 (18.4%)	159 (29%)	200 (36.5%)

### Socio-demographic, academic as well as substance-related factors associated with mental distress among Wollo university students.

Age, department choice, interest to one’s department, living area, cumulative GPA, frequency of attending worship, having close friend, family history of mental illness, using chat, smoking cigarette, alcohol use, and cannabis use within the past one month of data collection were found to have a *p*-value of < 0.25 in bivariate logistic regression analysis and thus fitted to multi-variable regression.

However, only never attending a place of worship (AOR = 4.2, 95% CI 1.73, 10.39), family history of mental illness (AOR = 2.1,95% CI 1.12, 3.95) current cigarette smoking (AOR = 3.2, 95% CI 1.69, 6.20), current alcohol use (AOR = 2.5,95% CI 1.49, 4.25), and current cannabis use (AOR = 3.4, 95% CI 1.18, 9.57) were found to be associated factors for mental distress with *p*-value < 0.05 in multi-variable analysis.

Even though, lack of interest to field of study (COR = 1.5, 95% CI 0.89, 2.67), having no close friend (COR = 1.5, 95% CI 0.94, 2.55), and current chat use (COR = 3.4, 95% CI 2.10, 5.43) revealed a higher odds of mental distress on bivariate analysis, this was not significant in multi-variable analysis. The multicollinearity diagnosis was done and Collinearity is not a problem of the current study as Variance inflation factor (VIF) is < 10 for all independent variables included in the multi-variable model (Table 4).

**Table 4:** A Bivariate and multi-variable Logistic Regression analysis table that shows the associations between mental distress and it’s socio-demographic and substance-related factors among Wollo university regular undergraduate students, Dessie, Ethiopia, 2019 (*n* = 548)

Independent variables	Category	Mental distress		COR (95% CI)	AOR (95% CI)
		Yes	No		
Age in years	Below mean (< 21)	50	155	*Reference*	*Reference*
	Above mean (≥ 21)	56	287	0.6 (0.39, 0.93)	0.52 (0.32, 0.85)
M89 Department of choice	Preferred	72	351	*Reference*	*Reference*
	Not preferred	34	91	0.55 (0.34, 0.88)	0.7 (0.35, 1.42)
CGPA	≥ 3.1	69	244	*Reference*	*Reference*
	≤ 3.00	37	198	0.66 (0.42, 1.03)	0.62 (0.38, 1.01)
Having close friends	Yes	79	362	*Reference*	*Reference*
	No	27	80	1.5 (0.94,2.55)	1.54 (0.88, 2.71)
Frequency of attending a place of worship	Daily	45	200	*Reference*	*Reference*
	2-3 times/week	17	127	0.59 (0.33, 1.08)	0.47 (0.24, 0.89)
	Once a week	16	54	1.32 (0.69, 2.51)	0.87 (0.43, 1.79)
	Less than a week	11	48	1.02 (0.49, 2.12)	0.69 (0.31, 1.54)
	Never	17	13	5.8 (2.63, 12.82)	4.2 (1.73, 10.39)**
Family hx of MI	Yes	22	48	2.2 (1.22, 3.75)	2.1 (1.12, 3.95)*
	No	84	394	*Reference*	*Reference*
Current cigarette smoking	Yes	32	30	5.9 (3.41, 10.36)	3.2 (1.69, 6.20)***
	No	74	412	*Reference*	*Reference*
Current alcohol use	Yes	48	92	3.0 (1.94, 4.74)	2.5 (1.49, 4.25)***
	No	59	350	*Reference*	*Reference*
Current cannabis use	Yes	13	9	6.7 (2.79, 16.19)	3.4 (1.18, 9.57)*
	No	93	433	*Reference*	*Reference*

*CGPA* cumulative grade point average, *Family hx of MI* family history of mental illness

Hosmer and lemshow goodness of fit test: chi-square = 3.85, df = 7 and *p*-value = 0.79

* Significant at *p*-value < 0.05 in multi-variable logistic regression

** Significant at *p*-value < 0.01 in multi-variable logistic regression

*** Significant at *p*-value < 0.001 in multi-variable logistic regression

## Discussion

To investigators' knowledge, this study is the first to assess the magnitude and associated factors of mental distress among university students in Ethiopia using the Kessler-10 item mental distress assessment scale. This might make it an important overview of magnitude as well as factors associated with mental distress in students in Ethiopia.

Out of 585 university students, 106 (19.3%); 95% CI (16.10, 22.80) had mental distress in this survey. At most 22.8% (upper bound of the 95% CI) of the university students included in this survey had suffered from mental distress within the 4 weeks before data collection. Considering severity, 65 (11.9%), 28 (5.1%), and 13 (2.4%) of study subjects were with mild, moderate, and severe mental distress, respectively.

A lower magnitude of mental distress was obtained in the current study as compared to a study conducted at Canadian university among medical students and residents, in which the prevalence of mental distress was found to be 39.7% (20.7% mild, 7.8% moderate, and 11.2% severe distress [12]. It is also lower than results of earlier studies which found that mental distress among university students was 50% in Singapore [13], 70% in Pakistan [14], 61.3% in Iraq [10] and 41% in Malaysia [16], 52% in India [17] and 26.9% in Nigeria [18] and 40.9%, 30%, and 39.6% in Ethiopia [19–21].

On the contrary, this study was higher than a study finding from Michigan; the USA in which only 15.6% of students have emotional distress [43] and 10.8% in Kenya [44]. The difference in prevalence between the current study and the above-mentioned studies might be due to differences in socio-economic, environmental, and behavioral risk factors. Besides, cultural factors in which mental illness is stigmatized by society in low-income countries like Ethiopia may hinder the help-seeking intention and practice of students, thus increasing the prevalence of mental distress. Also, most of the previous studies were conducted among medical students and it might be the stress full nature of medical study due to the high workload which increased the prevalence. Moreover, most of the previous studies used SRQ-20 and the current study used the Kessler-10 distress scale to screen mental distress and sample size variation might account for the difference in prevalence.

However, a result of this survey is consistent with studies in Australian universities (19.2%) [8] and 16.5% [15], 19.8% in Somaliland [30] and 21.6% in Ethiopia [28]. In the current study feeling that “everything was an effort”, feel restless/ fidgety and feel depressed was the mental distress symptoms complained most of the time by students within the past 4 weeks of study. This was more or less consistent with an Australian study at which the symptoms contributing most to mental distress were found to be feeling tired, ‘everything being an effort and being nervous [15].

In the current study, never attending a place of worship was a risk factor for mental distress. No matter how University students follow any type of religion, those students who never attend worship programs were 4.2 times more risky to developmental distress than students who attend worship programs daily. Supportive evidence for this was also found in studies from Adama [28] and Gondar [19]. The possible reason for this would be religion practice builds the highest strength in life and those with good religious practice will have good coping mechanisms from stressful events and factors [45, 46].

A family history of mental illness was also a determinant variable showing a positive association with mental distress in this study. Students who have a family member with mental illness were almost 2 times more likely to have mental distress as compared to those who have no family history of mental illness. These premises are supported by similar findings from studies conducted in Iraq [10], Pakistan [14], Adama [28], and Gondar [19]. Genetic predisposition, and the socio-economic as well as psychological burden caused by the mental illness of family members and care for it, might contribute to such a higher magnitude of mental distress [19].

In this study, current cigarette smoking, current alcohol use as well as current cannabis use were found to have a significant association with mental distress. University students included in current study and who were smoking cigarette, drinking alcohol and using cannabis within the past 4 weeks of study was 3.2, 2.5, and 3.4 times more likely to have a problem of mental distress as compared to those students who did not smoke cigarette, did not drink alcohol and did not use cannabis in the specified time period above respectively. Supportive findings for these were from a study in France [27], Nepal [29], Somaliland [30], and Gondar [19]. This might be due to multi-dimensional effects of substance use like a disturbance in the sleep–wake cycle, dependent producing effects of substances thus causing withdrawal, disturbing interpersonal relationships, increased class absence, and finally leading to poor academic achievement and development of mental distress [47]**.**

Even though, female sex was not found to have a significant association with mental distress in current study, previous studies in the United States [26], Iran [10], Sweden [31], Australia [8], Somaliland [30], and Gondar [19] showed an association between sex (being female) and mental distress in university students. The reason for this could be because, biologically females are predisposed to affective disorders, hormonal factors, and violence from environmental factors [48].

### Limitations

We should keep in mind the limitations of this study in analyzing, and utilizing its results. The primary limitation rose from the cross-sectional nature of study making causal inference difficult. So, further follow up studies that can solve this problem should be considered. Next, a lack of a dedicated instrument for the assessment of substance-related variables which are found to be associated factors for mental distress should be emphasized.

## Conclusion

One in five undergraduate regular students in Wollo University has been affected by mental distress as per the result of this study and never attending a place of worship, family history of mental illness, current cigarette smoking, current alcohol use as well as current cannabis use were its risk factors. Therefore, all stakeholders should emphasize this problem and strive to overcome it, since otherwise mental distress affects cognitive, emotional as well as interpersonal functioning and academic performance. Besides clubs regarding behavioral and substance-related issues should be established in the university and have to play an active role in bringing behavioral change to substance use.

## Data Availability

The datasets used throughout this research process are available from the corresponding author on a reasonable request.

## Abbreviations

AOR: adjusted odds ratio
CGPA: cumulative grade point average
OR: odds ratio
PHQ-9: Patient Health Questionnaire-9
SD: standard deviation
SPSS: Statistical Package for Social Science
SRQ-20: Self-reporting Questionnaire
USA: United States of America
WHO: World Health Organization
